# Oligonucleotide Enhancing Compound Increases Tricyclo-DNA Mediated Exon-Skipping Efficacy in the Mdx Mouse Model

**DOI:** 10.3390/cells12050702

**Published:** 2023-02-23

**Authors:** Flavien Bizot, Abdallah Fayssoil, Cécile Gastaldi, Tabitha Irawan, Xaysongkhame Phongsavanh, Arnaud Mansart, Thomas Tensorer, Elise Brisebard, Luis Garcia, Rudolph L Juliano, Aurélie Goyenvalle

**Affiliations:** 1 Université Paris-Saclay, UVSQ, Inserm, END-ICAP, 78000 Versailles, France; 2 Raymond Poincaré Hospital, APHP, 78266 Garches, France; 3 Medical Biology Department, Centre Scientifique de Monaco, Monaco 98000, Monaco; 4 LIA BAHN, CSM-UVSQ, Monaco 98000, Monaco; 5 Université Paris-Saclay, UVSQ, INSERM U1173, 2I, 78000 Versailles, France; 6 SQY Therapeutics, UVSQ, END-ICAP, 78000 Versailles, France; 7 INRAE Oniris, UMR 703 PAnTher, 44307 Nantes, France; 8 Initos Pharmaceuticals LLC, UNC Eshelman School of Pharmacy, University of North Carolina, Chapel Hill, NC 27599, USA

**Keywords:** antisense oligonucleotides, exon-skipping, endosomal escape, duchenne muscular dystrophy, UNC7938, tricyclo-DNA

## Abstract

Nucleic acid-based therapeutics hold great promise for the treatment of numerous diseases, including neuromuscular disorders, such as Duchenne muscular dystrophy (DMD). Some antisense oligonucleotide (ASO) drugs have already been approved by the US FDA for DMD, but the potential of this therapy is still limited by several challenges, including the poor distribution of ASOs to target tissues, but also the entrapment of ASO in the endosomal compartment. Endosomal escape is a well recognized limitation that prevents ASO from reaching their target pre-mRNA in the nucleus. Small molecules named oligonucleotide-enhancing compounds (OEC) have been shown to release ASO from endosomal entrapment, thus increasing ASO nuclear concentration and ultimately correcting more pre-mRNA targets. In this study, we evaluated the impact of a therapy combining ASO and OEC on dystrophin restoration in *mdx* mice. Analysis of exon-skipping levels at different time points after the co-treatment revealed improved efficacy, particularly at early time points, reaching up to 4.4-fold increase at 72 h post treatment in the heart compared to treatment with ASO alone. Significantly higher levels of dystrophin restoration were detected two weeks after the end of the combined therapy, reaching up to 2.7-fold increase in the heart compared to mice treated with ASO alone. Moreover, we demonstrated a normalization of cardiac function in *mdx* mice after a 12-week-long treatment with the combined ASO + OEC therapy. Altogether, these findings indicate that compounds facilitating endosomal escape can significantly improve the therapeutic potential of exon-skipping approaches offering promising perspectives for the treatment of DMD.

## 1. Introduction

Duchenne muscular dystrophy (DMD) is the most common muscular dystrophy in the paediatric population. It is caused by various pathogenic mutations in the DMD gene, which mostly disrupt the open reading frame and thus lead to an absence of functional dystrophin protein. The exon-skipping approach has been developed to restore the reading frame of the gene and is based on the use of antisense oligonucleotides (ASO). ASOs aim to eliminate one or several exons by masking key splicing sites and therefore induce the expression of an internally deleted but functional dystrophin protein. Several ASO-based drugs have already been approved by the US FDA for the treatment of DMD (eteplirsen, golodirsen, viltolarsen and casimersen targeting exons 51, 53 and 45) [1]. Approval was mostly based on safety, and a small percentage of dystrophin restoration was observed in muscle biopsies of treated patients. However, the clinical benefit is still marginal, and there is a critical need to improve the level of dystrophin being restored in DMD patients.

One of the most recognized challenges of ASO-mediated exon skipping is the delivery of ASOs to the nucleus of the target tissues [2,3]. To achieve this goal, second generations of ASO have been developed with alternative chemistries or various conjugates [4,5]. Amongst these, we have been working with one particular chemical substance named tricyclo-DNA (tcDNA), and we recently demonstrated that conjugation of palmitic acid to tcDNA significantly enhances its delivery to target tissues [6]. Despite these improvements, the quantity of ASO ending up in the nucleus to bind their pre-mRNA target remains extremely low. Indeed, even when ASOs reach the correct target tissues/cells, most of them end up trapped in the endosomal compartment and are degraded in the lysosomes before they can enter the cytoplasm and the nucleus (e.g., the ultimate target compartment) [2]. Previous studies have focused on ASO cellular trafficking to better characterize the mechanisms and improve the intracytoplasmic delivery of ASOs [3,7]. A number of studies have aimed at influencing ASO pharmacological effect by manipulating components of the endosomal machinery, specifically by targeting proteins involved in endomembrane trafficking, such as GCC2, M6PR, or COPII Golgi coat proteins [8,9,10].

Overall, these studies confirmed that components of the endosomal trafficking machinery are intimately involved in the subcellular fate and thus the pharmacological effectiveness of ASOs [11].

Several strategies have been considered to allow ASOs to cross the biological barriers and cell membranes. Among them, the conjugation of ASO to a cell penetration peptide (CPP) has been widely used. CPPs are relatively short peptides, from four to forty amino acids, which can induce endocytosis or promote the intracellular effects of ASO [12]. Given that CPPs are charged molecules, they are often conjugated with charge neutral ASOs, such as phosphorodiamidate morpholino oligomers (PMO) [13], and cannot be used with charged ASO like tcDNA. Other approaches based on the use of nanotechnology have been extensively studied, from DNA nanostructures to exosome-like nano-carriers to spherical nucleic acids or lipid nanoparticles (LNPs), all described in recent reviews [5,13,14]. An alternative option is to use small molecules named oligonucleotide enhancing compounds (OEC), which were discovered through high throughput screening and which selectively release oligonucleotides from non-productive entrapment in endosomal compartments [15,16,17]. Because they are not directly linked to the ASO, OECs may be used with all types of ASOs (charged or uncharged) to facilitate their access to the cytosol and thus improve the probability to reach the nucleus to substantially enhance pharmacological effects [11].

Different types of OECs have been characterized, depending on their mechanism of action: the first one, which has been known for decades is a “proton sponge action”, characteristic of lysosomotropic drugs, leads to an influx of water molecules, swelling, and disruption of the endosomal compartment [18]. Alternative mechanisms include disrupting the interaction with the proteins of the endosomal membrane (such as Retro-1, which affects Rab7/9 positive late endosomes) [19]. The more recently identified OEC, named UNC7938, was shown to selectively release oligonucleotides from late endosomes, with only little effect on lysosomal pH, thus emphasizing the distinction between the OECs and typical lysosomotropic compounds [11,17].

In this study, we report the impact of OEC UNC7938 administration on exon-skipping efficacy mediated by tcDNA-ASO in *mdx* mice and characterize the kinetics of the effect. Adult *mdx* mice were treated with a tcDNA-ASO aiming at skipping the *Dmd* exon 23 with or without UNC7938, and these were analyzed at different time points. The combined therapy appeared to be particularly efficient at early time points, as well as in the cardiac muscle, leading to a normalization of cardiac function after three months of treatment.

## 2. Materials and Methods

### 2.1. Antisense Oligonucleotides and Animal Experiments

Animal procedures were performed in accordance with national and European legislation, approved by the French government (Ministère de l’Enseignement supérieur et de la Recherche, Autorisation APAFiS #6518). Mdx (C57BL/10ScSc-Dmdmdx/J) mice were bred in our animal facility at the Plateforme 2Care, UFR des Sciences de la santé, Université de Versailles Saint Quentin and were maintained in a standard 12-h light/dark cycle with free access to food and water. Mice were weaned at week four to five (postnatal), and two to five individuals were housed per cage.

TcDNA-ASO, targeting the donor splice site of exon 23 of the mouse dystrophin pre-mRNA [20], was synthesized by SQY Therapeutics (Montigny le Bretonneux, France). Palmitic acid was conjugated at the 5′end of tcDNA-PO via a C6-amino linker and a phosphorothioate bond as previously described (Figure S1) [6]. Mice were injected intravenously with 30 mg/kg/wk of the tcDNA-ASO (one intravenous injection per week under general anesthesia using 2% isoflurane). The oligonucleotide enhancing compound UNC7938 was produced by Initos Pharmaceuticals LLC (Chapel Hill, NC, USA). *Mdx* mice were treated with UNC7938 at 15 mg/kg/wk under general anesthesia using 2% isoflurane 24 h after the ASO injection (every week or every four weeks, depending on the protocol). Age-matched *mdx* groups receiving an equivalent volume of sterile saline were included as controls, and C57BL/10 mice were included as wild-type controls.

Animals were euthanized at different time points according to the different protocols and muscles and tissues were harvested and snap-frozen in liquid nitrogen-cooled isopentane and stored at −80 °C before further analysis.

To assess the safety of UNC7938, liver and kidney were sampled at the end of the 12-wk protocol (two weeks after the last dose), fixed in 10% neutral buffered formalin, and embedded in paraffin wax. Thick sections of 4 µm were then routinely stained with hematoxylin-eosin-saffron (HES) for histopathological evaluation, which was further performed by a veterinary pathologist blind to treatment.

### 2.2. Echocardiography Procedure

The procedure was performed under isoflurane anesthesia. Anesthesia doses were kept to the lowest possible levels, usually 5% isoflurane for induction and 2.5–3% isoflurane during measurements. Animals were placed on a heating pad to maintain a constant body temperature (37 °C), and their rectal temperature was monitored throughout the experiment. Doppler-echocardiography was performed using a high-resolution ultrasound system (Logiq 9, GE, France) with a 36-MHz scan head. Each animal was shaven from the left sternal border to the left axillary line with depilatory cream before the examination. Each set of measurements was obtained from the same cardiac cycle. At least three sets of measurements were obtained from three different cardiac cycles. The left ventricular end-diastolic diameter (LVEDD), end-diastolic posterior wall thickness, and end-diastolic interventricular septal wall thickness were measured using the leading-edge convention of the American Society of Echocardiography from M mode. The LVEDD was measured, from a M-mode short-axis view of the left ventricle at the papillary muscle level. Left ventricular shortening fraction (RF) and left ventricular ejection fraction (LVEF) were calculated from the M mode. Aortic velocity time integral (VTI) was recorded during the procedure from Doppler echocardiography. Mitral inflow Doppler pattern was recorded (peak E, peak A, and deceleration time) from a four-chamber apical view. The left ventricular systolic intervals of the isovolumic contraction time (IVCT), the ventricular ejection time (ET), and the diastolic interval of the isovolumic relaxation time (IVRT) were measured for the Tei index calculation. Measurements were made for aortic and mitral blood flows recorded from an apical four-chamber modified view using pulsed Doppler, and the sample was placed between the tip of the mitral and the Left ventricular outflow tract. The Tei index was calculated as the ratio of (IVCT + IVRT) to systolic ejection time. Cardiac output (CO) was defined as stroke volume x heart rate. The shortening fraction (%) was calculated by the formula: (LVEDD-LVESD)/LVEDD × 100. LV end-diastolic (EDV) and end-systolic (ESV) volumes were calculated using a half ellipsoid model of the LV. From these volumes, LV ejection fraction (%) was calculated using the formula: (EDV-ESV)/EDV × 100. These experiments were performed in blind.

### 2.3. ASO Quantification by Fluorescent Hybridization Assay

Tissues were homogenized using the Precellys 24 (Bertin Instruments, Montigny le Bretonneux, France) at a final concentration of 50 mg/mL of lysis buffer (100 mmol/L Tris–HCl, pH 8.5, 200 mmol/L NaCl, 5 mmol/L EDTA, 0.2% sodium dodecyl sulfate) containing 2 mg/mL of proteinase K (Invitrogen, Germany) and incubated overnight at 55 °C in a hybridization oven. After centrifugation at 7000× *g* (Sorval ST 8R centrifuge, 75005719 rotor) for 15 min, the supernatant was used in the assay. Quantification of ASO was performed using a hybridization assay with a molecular beacon probe, as previously described [21].

### 2.4. Cell Fractionation and Western Blot Validation

Cell fractionation from freshly dissected gluteus muscles and protein extraction were performed as described previously (Dimauro et al., 2012). Then, 35 μg of protein were loaded onto 4–20% Mini-PROTEAN^®^ TGX™ Precast Protein Gels (BioRad, Carlsbad, CA, USA). The PVDF membranes were probed with primary polyclonal antibodies directed against histone H3 (Cell Signaling Technology, Danvers, MA USA, 1:1000) or primary monoclonal antibodies directed against EEA1 (Sigma Aldrich, Saint Louis, MI, USA, 1:1000) followed by incubation with a goat anti-mouse secondary antibody (IRDye 800 CW Goat anti-mouse IgG, Li-Cor, Germany, dilution 1/20,000) or goat anti-rabbit secondary antibody (IRDye 800 CW Goat anti-rabbit IgG, Li-Cor, Germany, dilution 1/20,000). Bands were visualized using the Odyssey CLx system (Li-Cor, Germany).

### 2.5. RNA Analysis

Total RNA was isolated from snap-frozen muscle tissues using TRIzol reagent, according to the manufacturer’s instructions (ThermoFisher Scientific, Carlsbad, CA USA). To visualize exon skipping levels, aliquots of 500 ng of total RNA were used for RT-PCR analysis using the Access RT-PCR System (Promega, Madison, WI, USA), as previously described [6].

Exon 23 skipping levels were also quantified using real-time quantitative PCR using Taqman assays designed against the exon 23–24 junction and exon 22–24 junction, as previously described [6].

### 2.6. Western Blot Analysis

Protein lysates were obtained from intervening muscle sections collected during cryosection using the Precellys 24 (Bertin Instruments, Montigny le Bretonneux, France) in RIPA buffer (ThermoFisher Scientific, Rockford, IL, USA) complemented with SDS powder (5% final) (Bio-Rad, Marnes-la-coquette, France) and protease inhibitor cocktail (ThermoFisher Scientific, Rockford, IL, USA). An amount of 25 μg of protein were loaded onto NuPAGE 3–8% Tris-Acetate Protein gels (Invitrogen, Carlsbad, CA, USA), following manufacturer instructions. Dystrophin protein was detected with NCL-DYS1 primary monoclonal antibody (NCL-DYS1; Novocastra, Newcastle, UK, dilution 1/200), as prevsiously described [6]. Quantification was performed using the Empiria Studio software (Li-Cor, Bad Homburg, Germany) based on a standard curve made from pooled lysates from C57BL10 (WT) and *mdx* control for each tissue.

### 2.7. Serum and Urine Analysis

Blood samples were collected at the end of the treatment for biochemistry analysis. Analyses of serum alanine aminotransferase (ALT), aspartate aminotransferase (AST), alkaline phosphatase (ALP), bilirubin, creatinine, urea, and albumin levels were performed by the pathology laboratory at Mary Lyon Centre, Medical Research Council, Harwell, Oxfordshire, UK.

Urine was collected using metabolic cages over 24 h, and urine creatinine and total protein were measured, as previously described [22].

### 2.8. Immunohistochemistry Analysis

Sections of 10 µm at 120 µm intervals were cut from triceps, diaphragm, and heart and examined for dystrophin expression using a rabbit polyclonal antibody Dystrophin (dilution 1:500; cat. number RB-9024-P ThermoScientific, Fremont, CA, USA), which was then detected by goat anti-rabbit IgGs Alexa Fluor 488 (dilution 1/500; A11070, Invitrogen, Eugene, OR, USA). Images were cropped, and scale bars of 100 µm were added using ImageJ software.

### 2.9. Statistical Analysis

All in vivo data were analyzed with the GraphPad Prism8 software (San Diego, CA, USA) and expressed as means ± S.E.M. The “*n*” refers to the number of mice per group.

Group comparisons were performed using one and two-way analyses of variance (ANOVA) with repeated-measure comparisons when needed (effects in different muscle tissues for example), followed by post-hoc Dunnett’s or Sidak’s multiple comparisons when appropriate. The Kruskal-Wallis test was used to compare groups that do not follow a normal distribution (assessed with the Shapiro-Wilk test). Significance levels were set at * *p* < 0.05, ** *p* < 0.01, *** *p* < 0.001, **** *p* < 0.0001.

## 3. Results

### 3.1. Effect of UNC7938 during a Short Term Exon Skipping Therapy in Mdx Mice

**Figure 1:**
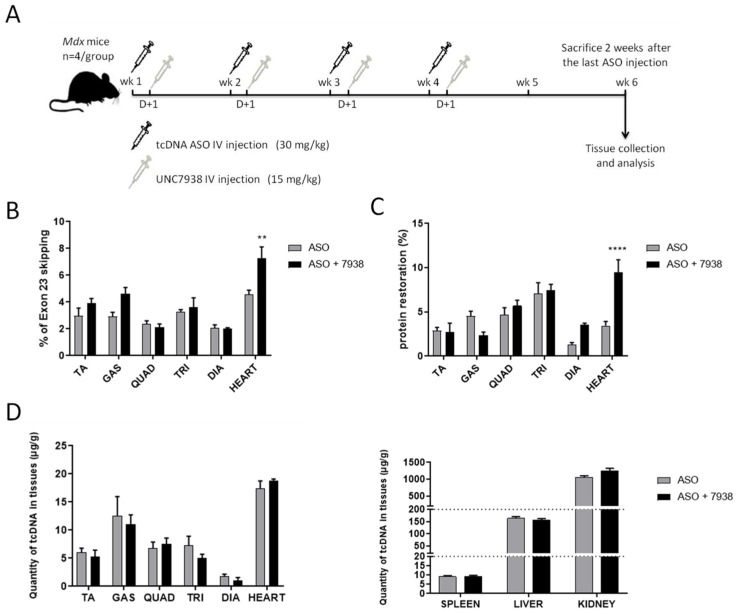
Short term efficacy of UNC7938 on exon skipping therapy in *mdx* mice. (**A**) Schematic representation of the injection protocol with tcDNA-ASO and the OEC UNC7938 in *mdx* mice. (**B**) Effect of UNC7938 on exon 23 skipping level. qPCR quantification of exon 23 skipping using taqman qPCR in the different muscle tissues. Tibialis anterior (TA), gastrocnemius (GAS), quadriceps (QUAD), triceps (TRI), diaphragm (DIA), and heart. *n* = 4 mice per group, ** *p* < 0.01 compared to ASO analyzed by RM two-way ANOVA. (**C**) Dystrophin restoration in treated *mdx* mice assessed by Western blotting. *n* = 4 mice per group, **** *p* < 0.0001 compared to ASO analyzed by RM two-way ANOVA. (**D**) Quantification of ASO in the different muscles tissues (left panel) and accumulation organs, such as spleen, liver, and kidney (right panel) after four weeks of treatment. Results are expressed as mean ± SEM; *n* = 4 mice per group.

We first studied the effects of the oligonucleotide enhancing compound (OEC) UNC7938 over a relatively short period of four weeks of treatment. In this protocol schematized in Figure 1A, *mdx* mice were treated for four weeks with the previously described palm-tcDNA-ASO targeting the *Dmd* exon 23, which has demonstrated an enhanced therapeutic index in this mouse model [6] and the OEC weekly. UNC7938 was administered intravenously at 15 mg/kg [23] 24 h after each ASO injection, and tissues were collected and analyzed two weeks after the last ASO injection (Figure 1A). Exon 23 skipping levels were quantified by RT-qPCR in several skeletal muscles, including tibialis anterior, gastrocnemius, quadriceps, triceps, and diaphragm, as well as the cardiac muscle. Results revealed an overall improvement of the ASO therapy with UNC7938 compared to tcDNA-ASO alone (*p* = 0.0152, RM two-way ANOVA, ASO vs. ASO + 7938) with a higher effect in the heart (*p* = 0.0016) (Figure 1B).

We next assessed the levels of dystrophin restoration in the different tissues and found higher dystrophin levels in mice treated with the ASO + OEC than in mice treated with the ASO alone (*p* = 0.0067, RM two-way ANOVA, ASO vs. ASO + 7938) with a particular strong effect in the heart reaching a 2.75 fold-increase (*p* < 0.0001) and in the diaphragm (2.75 fold-increase but not statistically significant *p* = 0.1680) (Figure 1C). The amount of tcDNA-ASO in the different muscles and off-target organs were measured, and we found that the overall content of ASO in tissue lysates was not impacted by UNC7938 treatment (Figure 1D) (treatment effect *p* = 0.6661 and 0.1183 for muscles and organs, respectively, analyzed by RM two-way ANOVA).

### 3.2. Kinetics of UNC7938 Effect on Exon Skipping Therapy in Mdx Mice

**Figure 2:**
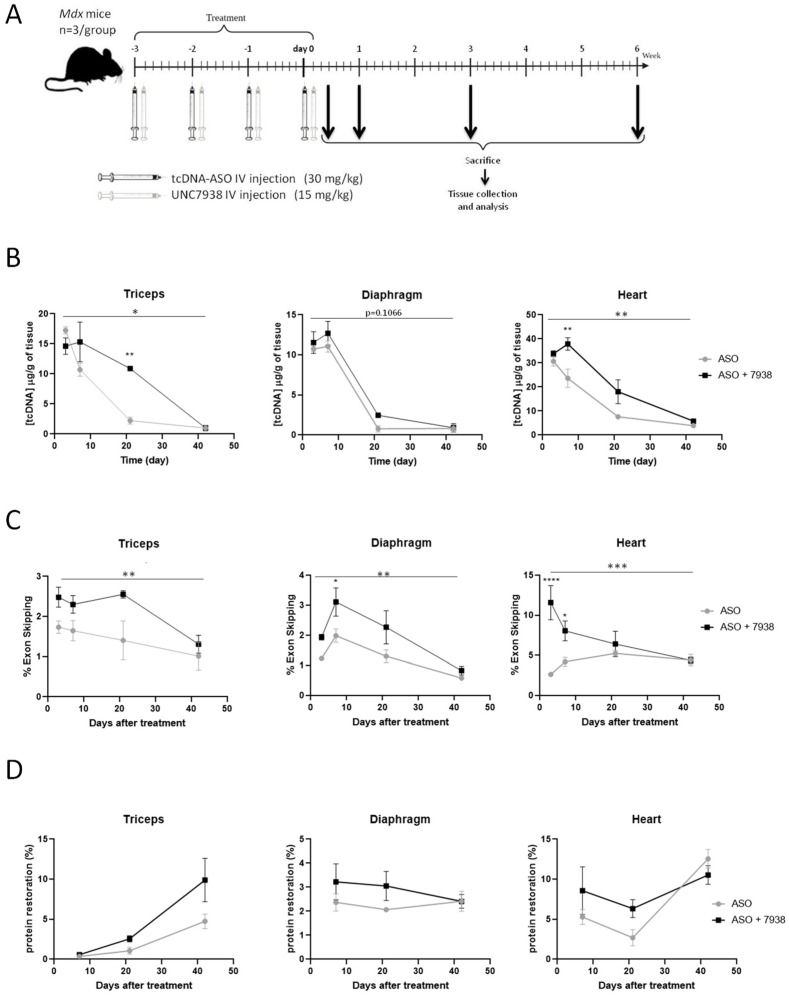
Kinetics of UNC7938 effect on exon skipping therapy in *mdx* mice. (**A**) Schematic representation of the injection protocol with tcDNA-ASO and the OEC UNC7938 in *mdx* mice with the different time points of analysis. (**B**) Quantification of ASO in triceps, diaphragm, and heart at different time points (72 h, one wk, three wks, and six wks) after the last ASO injection. *n* = 3 mice per group and per time point. * *p* < 0.05, ** *p* < 0.01 compared to ASO analyzed by two-way ANOVA. (**C**) Effect of UNC7938 on exon skipping level. qPCR quantification of exon 23 using taqman qPCR in the triceps, diaphragm, and heart at different time points (72 h, one wk, three wks and six wks) after last ASO injection. *n* = 3 mice per group and per time point. * *p* < 0.05, *** *p* < 0.001, **** *p* < 0.0001 compared to ASO analyzed by two-way ANOVA. (**D**) Quantification of dystrophin restoration levels by western blot in treated *mdx* mice at different time points (one wk, three wks, and six wks). Results are expressed as mean ± SEM; *n* = 3 mice per group and per time point.

To better characterize the effects of the OEC UNC7938 on ASO therapy, we treated *mdx* mice weekly with ASO + OEC during four weeks and analyzed them at different time points (72 h, one wk, three wks, and six wks) after the last injection (Figure 2A).

At very early time point (72 h), the ASO content in tissues was not impacted by UNC7938 administration in all muscles analyzed (Figure 2B and Figure S2A). At one-wk and three-wk time points, UNC7938 treatment appears to increase the ASO concentration in muscles, especially in the triceps (*p* = 0.0405) and in the heart (*p*= 0.0010). This difference suggests a slower elimination of the ASO in mice co-treated with the OEC compared to mice treated with ASO alone. However, six weeks after the last injection, ASO content was similar for both treatments and only limited amount of ASO were detected in all muscle tissues (Figure 2B and Figure S2A). We next measured exon skipping levels by RT-qPCR, and we found statistically higher levels of exon 23 skipping in muscles from mice treated with both ASO + OEC compared to mice treated with ASO alone. UNC7938 significantly enhanced exon skipping levels in heart (*p* = 0.0002 RM two-way ANOVA, ASO vs. ASO + 7938), with the strongest effect detected at early time points (*p* < 0.0001 at 72 h and *p* = 0.0321 at 1 wk). However, this difference decreased with time, since treatment with ASO alone induced a slower increase, though reaching similar levels as the combined therapy six weeks after the last injection (Figure 2C right). A similar effect was observed in the skeletal muscles (diaphragm: *p* = 0.0022, triceps: *p* =0.0048, quadriceps: *p* =0.0179, RM two-way ANOVA, ASO vs. ASO + 7938) (Figure 2C and Figure S2B).

We next assessed the levels of dystrophin expression in the various muscle tissues following treatment with ASO alone or ASO + 7938. At early time points (up to three weeks), UNC7938 tends to improve dystrophin restoration levels compared to ASO alone. For example, in the heart, the quantity of dystrophin produced is increased by 62% at one week and by 134% at three weeks. However, at later time points (six weeks), levels were similar in both groups of mice (Figure 2D and Figure S2C), making the overall difference (across all time points) not statistically significant (*p* = 0.5201 RM two-way ANOVA, ASO vs. ASO + 7938) (Figure 2D).

### 3.3. Combination of tcDNA-ASO and UNC7938 Efficiently Restores Dystrophin Expression and Improves Cardiac Function in Mdx Mice

Based on these promising results, in particular in the cardiac muscle, we aim to investigate a longer treatment period in order to assess some functional parameters, such as cardiac function. To lower the potential toxicity of repeated administrations of UNC7938 over three months, we wanted to reduce the number of UNC7938 injections, and we thus investigated whether one monthly injection would achieve similar effects than weekly injections. For that purpose, we performed an additional four-wk study in which UNC7938 was only administered after the fourth ASO injection. Analysis of exon skipping (Figure S3A) and protein restoration levels (Figure S3B) revealed no statistical difference between the two groups (ASO combined with one or four injections of OEC) with a *p* value of 0.4669 and 0.7915, respectively (analyzed by RM two-way ANOVA).

**Figure 3:**
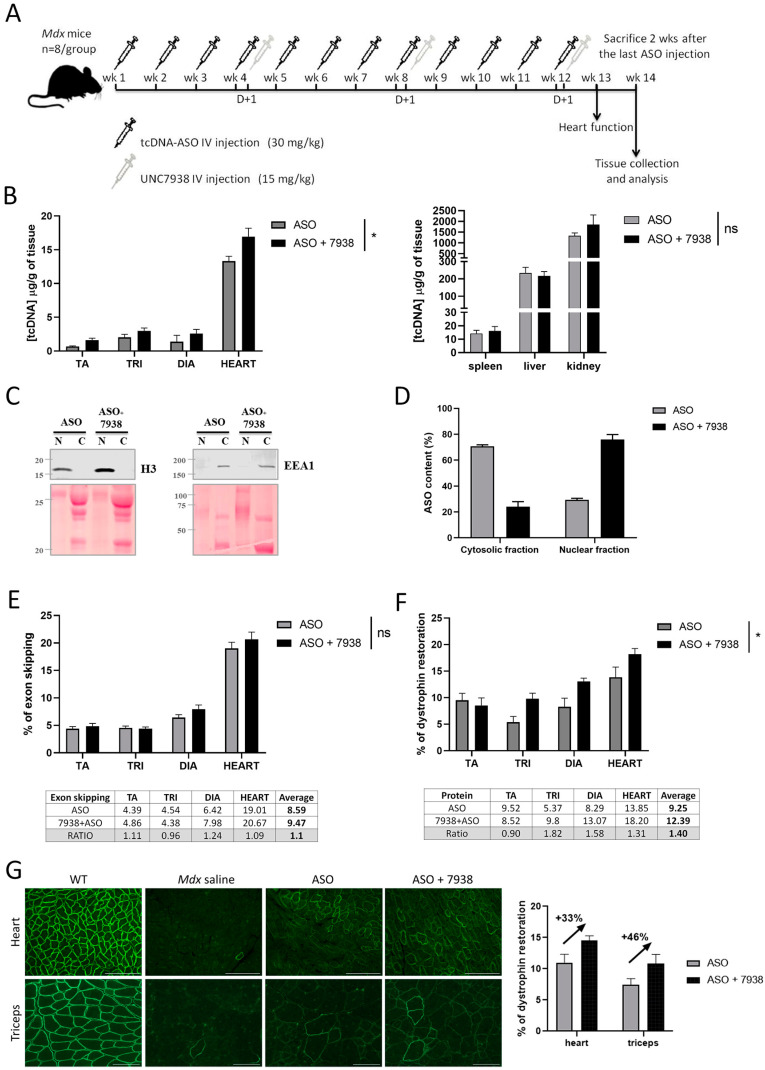
Efficacy of 12-wk combined UNC7938 and ASO treatment in *mdx* mice. (**A**) Schematic representation of the injection protocol with tcDNA-ASO and the OEC UNC7938 in *mdx* mice. (**B**) Quantification of ASO in the different muscles tissues (Tibialis anterior (TA), triceps (TRI), diaphragm (DIA) and heart) (left panel) and accumulation organs, such as spleen, liver and kidney (right panel) after 12 weeks of ASO treatment. *n* = 7 mice per group. (**C**) Subcellular fractionation and intracellular content of ASO following treatment with UNC7938. Western blot analysis of nuclear (*n*) and cytosolic (**C**) fractions isolated from gluteus muscles of *mdx* mice treated with ASO or ASO + 7938. The EEA1 and H3 antibodies are used to confirm cytosolic and nuclear enrichments, respectively. (**D**) Quantification of ASO in the cytosolic and nuclear fractions reveals a higher proportion of ASO in the nuclear fraction when mice have received the combined treatment ASO + 7938 compared to treatment with ASO alone. Results are expressed as means ± SEM; *n* = 6–8 mice per group, and two gluteus muscles are analyzed per mouse. (**E**) Effect of UNC7938 on exon skipping level. qPCR quantification of exon 23 using taqman qPCR in the different muscle tissues. *n* = 7 mice per group, (**F**) Dystrophin restoration assessed by Western blotting in treated *mdx* mice. Results are expressed as mean ± SEM; *n* = 7 mice per group. (**G**) Dystrophin staining in heart (top) and triceps (bottom). Detection of dystrophin protein (green staining) by immunofluorescence on transverse sections of muscle tissues (triceps and heart) from WT and *mdx* mice treated with saline, ASO, or ASO + UNC7938. Scale bar, 100 µm. Right panel: quantification of the dystrophin intensity staining in heart and triceps, mean fluorescence intensity is normalized to the number of fiber counts. Results are expressed as mean ± SEM; *n* = 4 mice per group. * *p* < 0.05 between ASO and ASO + 7938 analyzed by two-way ANOVA.

We, therefore, selected a monthly injection of UNC7938 for the long-term treatment investigating the therapeutic potential of the combination UNC7938 and tcDNA-ASO. Adult *mdx* mice were injected intravenously with 30 mg/kg/week of ASO and with 15 mg/kg/four weeks of UNC7938 over a period of 12 weeks (Figure 3A). ASO tissue concentrations were measured two weeks after the end of the treatment period in muscle tissues and off target organs. As previously observed in the kinetic study, we found slightly higher ASO amounts in muscles from mice co-treated with UNC7938 (*p* = 0.0142 RM two-way ANOVA, ASO vs. ASO + 7938), while there was no difference in spleen, liver, and kidney (*p* = 0.2704 RM two-way ANOVA, ASO vs. ASO + 7938) (Figure 3B). We hypothesized that these higher ASO contents in muscle lysates from mice treated with UNC7938 may result from increased amount of ASO reaching the nucleus, being less prone to elimination/degradation than the ASO fraction trapped in the endosome/lysosome compartments. To investigate this further, we sought to determine the ASO intracellular localization. Cell fractionation was performed on freshly isolated skeletal muscles from *mdx* mice treated with ASO alone or ASO + 7938 for three months, and we validated that the early endosome protein EEA1 was properly enriched in the cytosolic fraction, whereas the H3 histone was enriched in the nuclear fraction (Figure 3C). ASO content was determined in cytosolic and nuclear fractions, and the proportion of ASO detected in the nuclear fraction was higher in mice treated with ASO + 7938 compared to mice treated with ASO alone (Figure 3D).

We next evaluated the impact of UNC7938 on exon skipping and protein restoration levels in *mdx* mice treated for three months. Treatment with UNC7938 alone did not induce any exon skipping or dystrophin restoration (Figure S4). In mice treated with ASO + OEC, we found slightly, but not significantly, increased exon skipping levels in diaphragm and heart (*p* = 0.1928 RM two-way ANOVA, ASO vs. ASO + 7938) (Figure 3E) and significantly higher levels of dystrophin restoration compared to ASO alone (*p* = 0.03 RM two-way ANOVA, ASO vs. ASO + 7938) (Figure 3F). Dystrophin restoration was increased by 31% in the heart, 58% in the diaphragm, and 82% in the triceps (Figure 3F). Dystrophin expression and correct localization were also confirmed by immunostaining performed on muscle cryosections (Figure 3G). Analyses of mean fluorescence intensity revealed a similar increase in dystrophin restoration induced by UNC7938 co-treatment of approximately 33% and 46% in heart and triceps, respectively.

**Figure 4:**
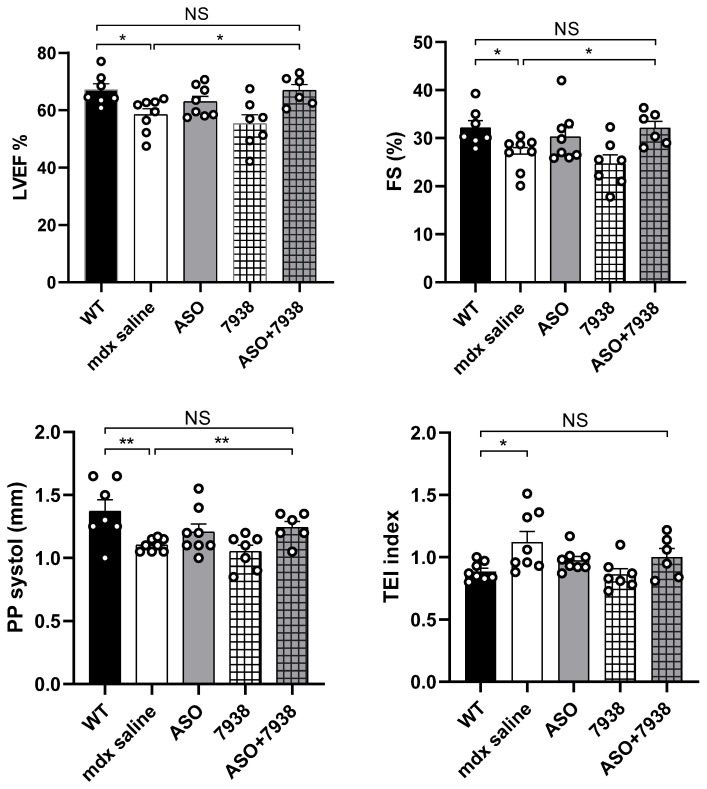
The combination of tcDNA-ASO and UNC7938 treatment improves cardiac function in *mdx* mice. Cardiac function was evaluated by echocardiography in six-month-old mice and the left ventricular ejection fraction (LVEF), the fractional shorting (FS), the systolic pulse pressure (PP systole), and the Tei index are represented. Results are expressed as mean ± SEM; *n* = 8 for WT, *mdx* saline and ASO, *n* = 7 for 7938 and *n* = 6 for ASO + 7938. * *p* < 0.05, ** *p* < 0.01 analyzed by one-way ANOVA.

Since cardiomyopathy is a typical feature of DMD and that the combined ASO + 7938 treatment appears to be particularly efficient in the heart, we wanted to investigate cardiac function outcomes after these three months of treatment. Echocardiography in six-month-old *mdx* mice revealed a significant reduction of the left ventricular ejection fraction (LVEF), the fractional shortening (FS), and the systolic pulse pressure (PP systole), as well as an increase in the Tei index compared with WT mice (Figure 4). Treatment of *mdx* mice with 30 mg/kg/wk of tcDNA-ASO for 12 weeks improved all parameters, although the differences did not reach statistical significance (*p* > 0.05 analyzed by one-way ANOVA). After co-treatment with UNC7938 however, LVEF, FS, and PP systole were significantly restored (*p* = 0.0154, 0.0153 and 0.0097 compared with *mdx* saline for LVEF, FS, and PP systole, respectively, one-way ANOVA) and no different from WT mice, indicating a normalization of heart function. Only the Tei index was still different between *mdx* control and ASO + 7938 treated mice (*p* = 0.3161), but it was no longer different from the WT mice (*p* value = 0.0951), demonstrating a promising improvement.

### 3.4. Safety Assessment of the Combined Treatment with UNC7938 and tcDNA-ASO

**Figure 5:**
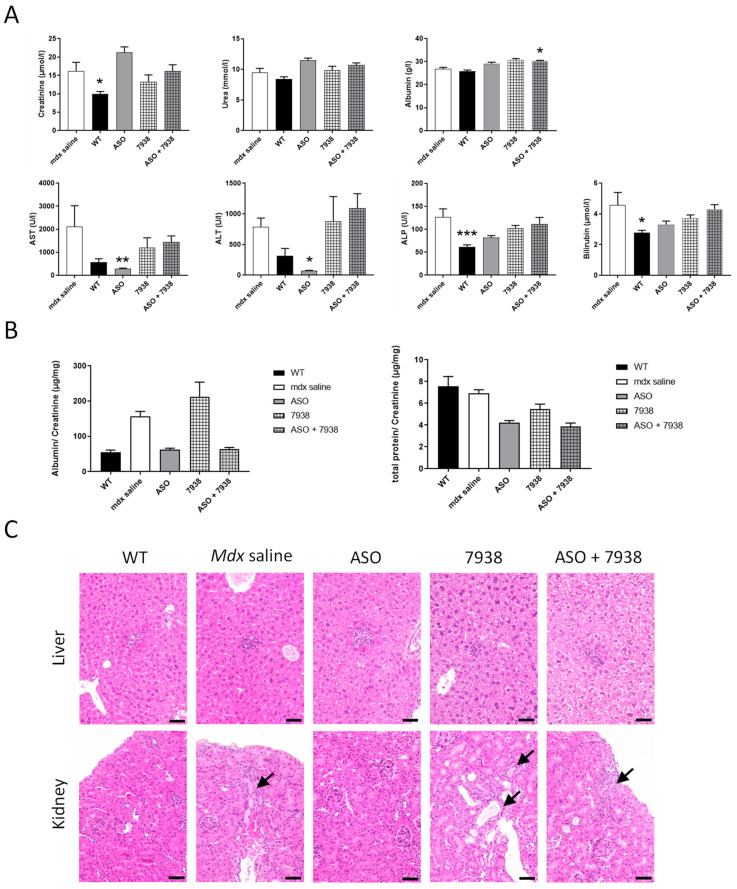
Safety profile of the combined UNC7938 + ASO treatment. (**A**) Quantification of general toxicity biomarkers in the serum: creatinine, urea, albumin, aspartate aminotransferase (AST), alanine aminotransferase (ALT), alkaline phosphatase (ALP), and bilirubin. Results are expressed as mean ± SEM; *n* = 5–8 mice per group, * *p* < 0.05, ** *p* < 0.01, *** *p* < 0.001 compared to *mdx* saline, analyzed by Kruskal-Wallis one-way ANOVA. (**B**) Quantification of total protein and albumin levels in the urine of WT and treated *mdx* mice. Urine was collected at the end of the 12-wk treatment. Results are normalized to creatinine levels and expressed as mean ± SEM; *n* = 5–8 mice per group; *p* = ns compared to *mdx* saline (one-way ANOVA). (**C**) Histological presentation of wild-type mice and *mdx* mice treated with saline, ASO, UNC7938 or ASO + 7938 for 12 weeks. In liver (upper panel), small foci of inflammatory cell infiltration were scattered in the hepatic parenchyma of all mice in every group. In kidney (lower panel), no lesions or only sporadic changes were observed except for two animals: one UNC7938-treated mouse and one ASO + 7938-treated mouse displayed few minimal areas of tubular degeneration/regeneration (arrows) +/− associated with intraluminal proteinaceous casts and tubular ectasia. Note that one similar area was observed in one *mdx*-saline mouse. Hematoxylin-Eosin-Saffron staining. Scale bar = 50 µm.

To investigate whether the combination of UNC7938 and tcDNA-ASO induced any potential safety signals, we first analyzed the serum levels of various biomarkers in mice following the different treatments. Quantification of serum creatinine, urea, ALP, bilirubin, and transaminases (ALT and AST) revealed no significant increases in UNC7938 treated *mdx* mice compared to saline treated *mdx* mice (Figure 5A). Only albumin was found to be slightly increased with the combined therapy ASO + 7938. We next evaluated some urinary biomarkers, including albumin and total protein, and found no significant changes in *mdx* mice treated or co-treated with UNC7938.

Finally, we explored the histopathological profile of liver and kidneys (the two main sentinel organs for drug toxicity) in treated *mdx* mice and WT controls in order to evaluate potential adverse effects of UNC7938, alone or in combined therapy (Figure 5C). In the liver, all mice of every groups (WT, *mdx* saline, *mdx* ASO, *mdx* UNC7938, and *mdx* ASO + 7938) similarly displayed some small scattered foci of inflammatory cell infiltration, sometimes associated with few individual necrotizing hepatocytes. Foci of inflammatory cell infiltration is a common background lesion in the liver of mice. The only other histopathological change observed was very small amount of pigment in Kupffer cells and, less frequently, in Ito cells. This was observed in all animals treated with ASO (ASO alone and combined with 7938). Such a small degree of pigment, without associated cellular reaction or lesion, is generally considered biologically irrelevant. In the kidney, most of the mice displayed no lesion or only unspecific sporadic changes (proteinaceous casts, altered glomeruli, basophilic tubules). Only one UNC7938-treated mouse (out of 7) and one ASO + 7938-treated mouse (out of six) displayed more pronounced lesions than what observed in WT mice and *mdx*-saline-treated mice, consisting of few foci of tubular degeneration and regeneration, sometimes associated with intraluminal proteinaceous casts. Those lesions, very minimal, unspecific, and present in a very low number of animals, were not considered compound-related and significant. Thus, no hepatic or renal toxicity has been associated with UNC7938 when administrated intravenously at the dose of 15 mg/kg/4 week over a period of 12 weeks, alone or in combination with tcDNA-ASO. These findings suggest that the increased effect of ASO observed with OEC unlikely results from toxicity of UNC7938.

## 4. Discussion

In this study, we aimed to enhance the therapeutic potential of ASO-mediated exon skipping for DMD, which is still currently limited by several challenges. Our previous work has focused on improving the ASO compound itself, and we have optimized a tcDNA-based ASO, free of phosphorothioate linkages and conjugated to a palmitic acid, presenting a significantly higher therapeutic index [6]. Besides the optimization of the ASO compound itself, which may address some of the delivery issues, it is possible to act on cellular mechanisms to facilitate the transport of ASO to their target organelle, i.e., the nucleus in the case of splice switching ASO. The inefficiency of endosomal escape is considered one of the biggest problem preventing the widespread use of RNA therapeutics [24]. The chemical manipulation of the endosome trafficking machinery has been shown to positively impact ASO delivery and pharmacological effects [11]. However, the proof of concept has never been demonstrated for DMD and the exon skipping approach in vivo. In the present study, we therefore evaluated the potential of such a combined therapy in vivo in the *mdx* mouse model of DMD. We selected the UNC7938 compound that was discovered through high throughput screening and shown to selectively release ASO from non-productive entrapment in endosomal compartments [15,16,17]. UNC7938 was recently shown to potentiate the effect of a peptide conjugated PMO (PPMO) in cystic fibrosis patients cells and in vivo in mouse lung [23], but no data are available in muscle tissues.

In our preliminary four-wk treatment protocol, we showed that the combined ASO + UNC7938 therapy induced significantly higher exon skipping level (1.59 fold-changes) and dystrophin restoration levels (2.75 fold-changes) than ASO therapy alone, especially in the heart. This is in line with our hypothesis that UNC7938 treatment may help ASO to escape the endosome and diffuse to the nucleus where the pre-mRNA targets are located. However, the reasons underlying the higher effect in the heart in contrast with the other muscle tissues remain unclear and may simply be due to the fact that tcDNA is particularly efficient at targeting the heart (i.e., heart is the muscle with the highest content of ASO).

To better characterize the effects of UNC7938, we studied its impact at different time points after injection. The quantification of ASO in different tissues shows that ASO distribution is not affected by UNC7938 at the very early time point (72 h), suggesting that UNC7838 does not impact the distribution of ASO to the different tissues per se. However, ASO tissue content appeared higher between one and three weeks post-treatment in UNC7938 co-treated mice compared to mice treated with ASO alone. This suggests that ASOs are not eliminated as fast when the OEC is administrated, which would be in line with their mechanism of action. Endosomal escape would protect the ASO from a quick degradation/elimination. This was also confirmed by the pharmacological effect of ASO, which was significantly enhanced after the treatment with UNC7938. The effect was once again more important in the heart, which is the muscle tissue with the highest content of ASO, with up to a 4.4-increase in exon skipping levels at early time points. Interestingly, this ‘booster’ effect faded over time, and ASO content, as well as exon skipping levels, ended up being very similar six weeks after the treatment in both groups of mice (with or without UNC7938). This may be explained by the ‘depot effect’ occurring in mice treated with ASO alone, in which the ASO trapped in endosomes are actually slowly released over time. This phenomenon characterized by the fact that ~99% of ASO (or RNA therapeutics in general) are typically trapped inside endosomes, may indeed explain why ASO can achieve long pharmacological effects due to a constant low level of leakage into the cytoplasm over weeks or months [24]. Using an OEC, such as UNC7938, may induce a short liberation of ASO from the endosomes, but might, in counterpart, affect the depot effect, which would no longer occur. This could explain why the overall exon skipping levels end up being similar several weeks after the treatment.

We next investigated the impact of UNC7938 on exon skipping therapy over a longer period of time and treated *mdx* mice for 12 weeks, during which they received three injections of UNC7938 (every four weeks). We had previously validated that this dosing regimen induces similar effects that an injection every week. The ASO content in muscle tissues at the end of the 12-wk treatment was slightly higher in mice co-treated with UNC7938 compared to mice treated with ASO alone, as previously observed. In order to determine the ASO intracellular localization, we performed cell fractionation on freshly isolated skeletal muscles from treated *mdx* mice and quantified the ASO content in enriched nuclear and cytoplasmic fractions. Interestingly, we found a higher proportion of ASO in the nuclear fraction of mice treated with ASO + UNC7938 compared to mice treated with ASO alone, which showed a higher proportion of ASO in the cytosolic fraction. We found approximately 71% of ASO in the cytosolic fractions in mice treated with ASO alone, which is higher that the commonly assumed 99% of ASO trapped in endosomes [24]. This may be due to the repeated injections of ASO over 12 weeks, which may help accumulate more ASO in the nucleus than single injections, and also to the timing of analysis. Since muscles were analyzed two weeks after the last injection, a proportion of ASO that was trapped in the endosomal/lysosomal compartment may have already been cleared, thus lowering the quantified cytoplasmic fraction compared to the nuclear fraction. It would be interesting to perform similar fractionation analysis 24 h after a single injection as this may reveal different proportions. However, one should also keep in mind that cell fractionation protocol results in an enrichment of fractions rather that absolutely pure fractions, so it cannot be excluded that the ASO content in the nuclear fraction is slightly overestimated because of traces of cytosolic fraction. Nonetheless, the consistent higher proportion of ASO found in the nuclear fraction after UNC7938 treatment is in line with its mechanism of action and confirms that freeing ASO from endosomes helps them reach their ultimate target organelle, the nucleus.

The exon skipping levels and restoration of dystrophin protein were also slightly improved in co-treated mice, although to a lesser extent than after the four-wk treatment. This may be explained by the depot effect that would be more noticeable after several weeks. In mice treated with ASO alone, because most of ASO are trapped in endosomes and only slowly released over time, the pharmacological effects may be delayed compared to mice co-treated with UNC7938 in which the released ASO can act more rapidly after the injection.

Considering the clear increase in the amount of ASO detected in the nuclear fraction after treatment with UNC7938, the effect reported on exon skipping levels and dystrophin rescue may appear relatively low. This may be explained by the limited amount of ASO that overall reached the target tissues. Indeed, despite improvement in the design of our tcDNA ASO, conjugated to palmitic acid in order to increase its biodistribution to muscle tissues, the proportion of ASO actually reaching skeletal muscles remains low, as shown in Figure 3B (between 0.6 and 3 µg/g of tissues). This highlights an important consideration to keep in mind—that the full potential of endosomal escape compounds can only be achieved in combination with efficient delivery of ASO to the target tissue.

Nonetheless, co-treatment of ASO + UNC7938 led to higher dystrophin restoration than treatment with ASO alone, especially in the heart (+31%), the diaphragm (+58%), and the triceps (+82%). This was also confirmed by immunostaining, which revealed a correct localization of dystrophin at the subsarcolemmal space of muscle fibers and higher intensity of staining. Given the particularly strong effect observed in the heart with UNC7938 and considering the importance of cardiac dysfunction in DMD patients, we investigated the cardiac function in treated *mdx* mice. *Mdx* mice typically show a progressive development of cardiac defects from six months of age [25,26], and electrocardiography investigations in the control *mdx* mice from this study indeed revealed significant increase in the Tei index, as well as a reduction of the systolic pulse pressure (PP systole) of the left ventricular ejection fraction (LVEF) and of the fractional shortening (FS) compared with WT mice. We found that treatment with ASO alone improved all these parameters, although the difference did not reach statistical significance. It should be noted that we demonstrated, in a previous study, that treatment with tcDNA-ASO alone can significantly improve cardiac function in *mdx* mice after 12 weeks of treatment at 50 mg/kg/wk [6]. In the current study, we selected a lower dose of 30 mg/kg/wk on purpose to be able to detect differences with the UNC7938 co-treated mice. Remarkably, *mdx* mice treated with both tcDNA-ASO and UNC7938 presented statistically significant improvement in all parameters, even reaching WT values (non-statistical difference from WT values for all parameters analyzed). This improved effect over the treatment with ASO alone at this dose likely results from higher dystrophin restoration levels in the heart of co-treated mice, but also from a possible earlier restoration. Indeed, as mentioned previously, one of the advantages of OEC molecules is their capacity to quickly release ASO from endosomes and therefore allow ASO to act more rapidly than those which would be slowly released through the depot effect. This may play an important role for cardiac function, which is more likely to be normalized when dystrophin expression has been restored for longer.

All together, our data confirm the potential of OECs, such as UNC7938, to enhance the therapeutic index of exon-skipping ASO for DMD. Yet, this may counterbalance the depot effect, which classically allows long-term effect of ASO. Ultimately, one should aim at keeping some depot effect while releasing sufficient ASO for rapid effect. Considering the high amount of ASO trapped in endosome compartments, believed to be around 99%, freeing up to 50% would suffice to maintain both rapid and long term effects.

Overall, this work establishes the proof of concept that using small molecules facilitating ASO endosomal escape enhances their efficacy in a DMD mouse model, thus opening new therapeutic avenues for combined therapies.

## Data Availability

The primary data for this study are available from the authors upon request.

## Supplementary Materials

The following supporting information can be downloaded at: https://www.mdpi.com/article/10.3390/cells12050702/s1, Figure S1: Representation of the tcDNA-ASO used in this study, Figure S2: kinetic efficacy of UNC7938 on exon skipping therapy in *mdx* mice, Figure S3: Comparison between 1 vs. 4 injections of UNC7938 on exon skipping efficacy, Figure S4: Treatment with UNC7938 alone has no effect on dystrophin restoration.

## References

[1] Fortunato F., Rossi R., Falzarano M., Ferlini A. (2021). Innovative Therapeutic Approaches for Duchenne Muscular Dystrophy. J. Clin. Med..

[2] Crooke S.T., Wang S., Vickers T.A., Shen W., Liang X.-H. (2017). Cellular Uptake and Trafficking of Antisense Oligonucleotides. Nat. Biotechnol..

[3] Ono D., Asada K., Yui D., Sakaue F., Yoshioka K., Nagata T., Yokota T. (2020). Separation-related rapid nuclear transport of DNA/RNA heteroduplex oligonucleotide: Unveiling distinctive intracellular trafficking. Mol. Ther. Nucleic Acids.

[4] Ferlini A., Goyenvalle A., Muntoni F. (2021). RNA-Targeted Drugs for Neuromuscular Diseases. Science.

[5] Roberts T.C., Langer R., Wood M.J.A. (2020). Advances in oligonucleotide drug delivery. Nat. Rev. Drug Discov..

[6] Relizani K., Echevarría L., Zarrouki F., Gastaldi C., Dambrune C., Aupy P., Haeberli A., Komisarski M., Tensorer T., Larcher T. (2021). Palmitic acid conjugation enhances potency of tricyclo-DNA splice switching oligonucleotides. Nucleic Acids Res..

[7] Juliano R.L. (2018). Intracellular Trafficking and Endosomal Release of Oligonucleotides: What We Know and What We Don’t. Nucleic Acid Ther..

[8] Liang X.-H., Sun H., Shen W., Crooke S.T. (2015). Identification and characterization of intracellular proteins that bind oligonucleotides with phosphorothioate linkages. Nucleic Acids Res..

[9] Liang X.-H., Sun H., Hsu C.-W., Nichols J.G., A Vickers T., De Hoyos C.L., Crooke S.T. (2019). Golgi-endosome transport mediated by M6PR facilitates release of antisense oligonucleotides from endosomes. Nucleic Acids Res..

[10] Liang X.-H., Sun H., Nichols J.G., Allen N., Wang S., Vickers T.A., Shen W., Hsu C.-W., Crooke S.T. (2018). COPII vesicles can affect the activity of antisense oligonucleotides by facilitating the release of oligonucleotides from endocytic pathways. Nucleic Acids Res..

[11] Juliano R.L. (2021). Chemical Manipulation of the Endosome Trafficking Machinery: Implications for Oligonucleotide Delivery. Biomedicines.

[12] Langel Ü. (2021). Cell-Penetrating Peptides and Transportan. Pharmaceutics.

[13] Hammond S.M., Aartsma-Rus A., Alves S., Borgos S.E., Buijsen R.A.M., Collin R.W.J., Covello G., Denti M.A., Desviat L.R., Echevarría L. (2021). Delivery of oligonucleotide-based therapeutics: Challenges and opportunities. EMBO Mol. Med..

[14] Gagliardi M., Ashizawa A.T. (2021). The Challenges and Strategies of Antisense Oligonucleotide Drug Delivery. Biomedicines.

[15] Juliano R.L., Wang L., Tavares F., Brown E.G., James L., Ariyarathna Y., Ming X., Mao C., Suto M. (2018). Structure–activity relationships and cellular mechanism of action of small molecules that enhance the delivery of oligonucleotides. Nucleic Acids Res..

[16] Wang L., Ariyarathna Y., Ming X., Yang B., James L.I., Kreda S.M., Porter M., Janzen W., Juliano R.L. (2017). A Novel Family of Small Molecules that Enhance the Intracellular Delivery and Pharmacological Effectiveness of Antisense and Splice Switching Oligonucleotides. ACS Chem. Biol..

[17] Yang B., Ming X., Cao C., Laing B., Yuan A., Porter M.A., Hull-Ryde E.A., Maddry J., Suto M., Janzen W.P. (2015). High-throughput screening identifies small molecules that enhance the pharmacological effects of oligonucleotides. Nucleic Acids Res..

[18] Bus T., Traeger A., Schubert U.S. (2018). The great escape: How cationic polyplexes overcome the endosomal barrier. J. Mater. Chem. B.

[19] Ming X., Carver K., Fisher M., Noel R., Cintrat J.-C., Gillet D., Barbier J., Cao C., Bauman J., Juliano R.L. (2013). The small molecule Retro-1 enhances the pharmacological actions of antisense and splice switching oligonucleotides. Nucleic Acids Res..

[20] Relizani K., Griffith G., Echevarría L., Zarrouki F., Facchinetti P., Vaillend C., Leumann C., Garcia L., Goyenvalle A. (2017). Efficacy and Safety Profile of Tricyclo-DNA Antisense Oligonucleotides in Duchenne Muscular Dystrophy Mouse Model. Mol. Ther. Nucleic Acids.

[21] Echevarria L., Aupy P., Relizani K., Bestetti T., Griffith G., Blandel F., Komisarski M., Haeberli A., Svinartchouk F., Garcia L. (2019). Evaluating the Impact of Variable Phosphorothioate Content in Tricyclo-DNA Antisense Oligonucleotides in a Duchenne Muscular Dystrophy Mouse Model. Nucleic Acid Ther..

[22] Zhang A., Uaesoontrachoon K., Shaughnessy C., Das J.R., Rayavarapu S., Brown K.J., Ray P.E., Nagaraju K., Anker J.N.V.D., Hoffman E.P. (2015). The use of urinary and kidney SILAM proteomics to monitor kidney response to high dose morpholino oligonucleotides in the mdx mouse. Toxicol. Rep..

[23] Dang Y., van Heusden C., Nickerson V., Chung F., Wang Y., Quinney N.L., Gentzsch M., Randell S.H., Moulton H.M., Kole R. (2021). Enhanced delivery of peptide-morpholino oligonucleotides with a small molecule to correct splicing defects in the lung. Nucleic Acids Res..

[24] Dowdy S.F., Setten R.L., Cui X.-S., Jadhav S.G. (2022). Delivery of RNA Therapeutics: The Great Endosomal Escape!. Nucleic Acid Ther..

[25] Quinlan J.G., Hahn H.S., Wong B.L., Lorenz J.N., Wenisch A.S., Levin L.S. (2004). Evolution of the mdx mouse cardiomyopathy: Physiological and morphological findings. Neuromuscul. Disord..

[26] Spurney C.F., Knoblach S., Pistilli E.E., Nagaraju K., Martin G.R., Hoffman E. (2008). Dystrophin-deficient cardiomyopathy in mouse: Expression of Nox4 and Lox are associated with fibrosis and altered functional parameters in the heart. Neuromuscul. Disord..

