# Supplementary material for: Oligonucleotide Enhancing Compound Increases Tricyclo-DNA Mediated Exon-Skipping Efficacy in the Mdx Mouse Model
Source: Cells. 2023 Feb 23;12(5):702. doi: 10.3390/cells12050702 (PMC10001065; doi:10.3390/cells12050702)
Supplement: Supplementary file 1 [file cells-12-00702-s001.zip › cells-2178082-supplementary.pdf]

cells

MDPI

Supplementary material

Oligonucleotide enhancing compound increases tricyclo-DNA mediated exon-skipping efficacy in the mdx mouse model

Flavien Bizot[1], Abdallah Fayssoil[1,2], Cécile Gastaldi[3], Tabitha Irawan[1], Xaysongkhame Phongsavanh[1], Arnaud Mansart[4], Thomas Tensorer[5], Elise Brisebard[6], Luis Garcia[1], Rudolph L Juliano[7], and Aurélie Goyenvalle[1,*]

[1] Université Paris-Saclay, UVSQ, Inserm, END-ICAP, 78000 Versailles, France
[2] Raymond Poincaré Hospital, APHP, Garches, France.
[3] Centre Scientifique de Monaco, LIA-BAHN, Monaco, Monaco
[4] Université Paris-Saclay, UVSQ, INSERM U1173, 2I, Montigny-le-Bretonneux, France
[5] SQY Therapeutics, UVSQ, END-ICAP, 78180 Montigny le Bretonneux, France.
[6] INRAE Oniris, UMR 703 PAnTher, Nantes, France
[7] Initos Pharmaceuticals LLC, UNC Eshelman School of Pharmacy, University of North Carolina, Chapel Hill, North Carolina
* Correspondence: aurelie.goyenvalle@uvsq.fr; Tel.: +33170429432

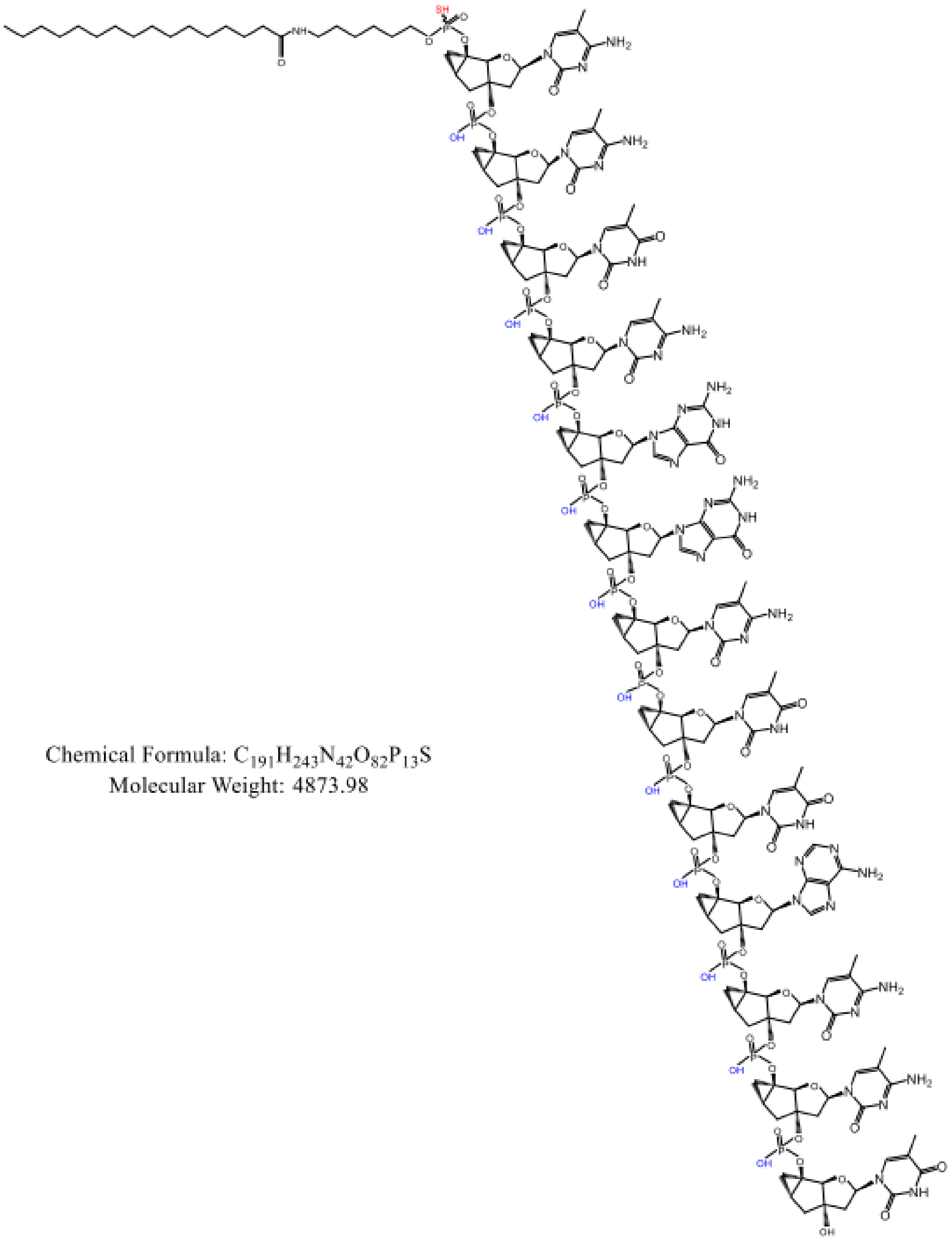

Chemical Formula: $C_{191}H_{243}N_{42}O_{82}P_{13}S$
Molecular Weight: 4873.98

Figure S1. Representation of the tcDNA-ASO targeting the donor splice site of exon 23 of the mouse dystrophin pre-mRNA used in this study. Palmitic acid is conjugated at the 5′end of tcDNA-PO via a C6-amino linker and a phosphorothioate bond.

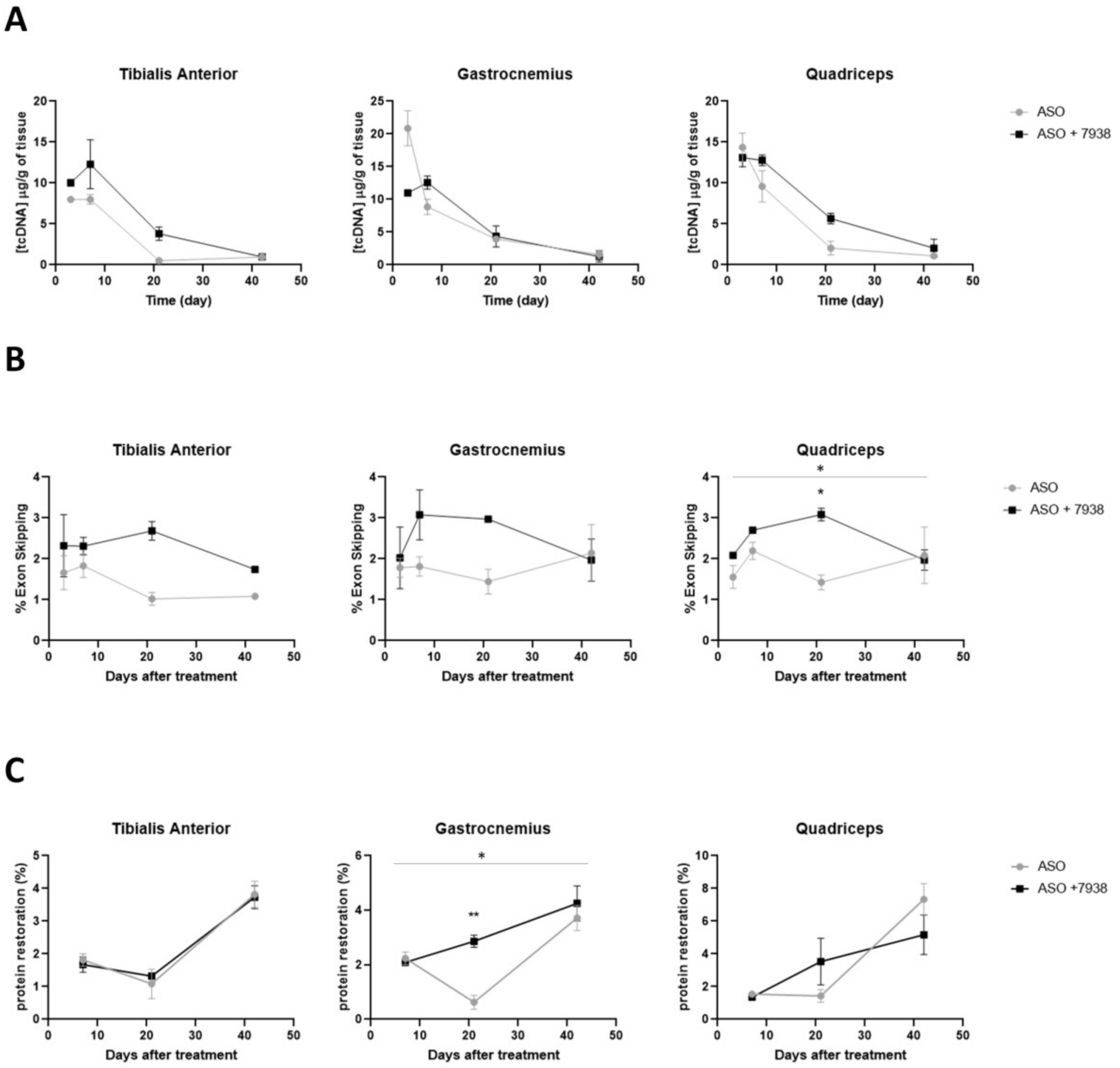

Figure S2. kinetic efficacy of UNC7938 on exon skipping therapy in *mdx* mice. (A) Quantification of ASO in tibialis anterior, gastrocnemius and quadriceps at different time points (72h, 1 week, 3 weeks and 6 weeks) after the last ASO injection. N=3 mice per group and per time point. (B) Effect of UNC7938 on exon skipping level. qPCR quantification of exon 23 skipping using taqman qPCR in the tibialis anterior, gastrocnemius and quadriceps at different time points (72h, 1 week, 3 weeks and 6 weeks) after last ASO injection. N=3 mice per group and per time point. *p<0.05, compared to ASO analyzed by two-way ANOVA. (C) Quantification of dystrophin restoration levels in treated mdx mice at different time points (1 week, 3 weeks and 6 weeks). N=3 mice per group and per time point. *p<0.05, **p<0.01 compared to ASO analyzed by two-way ANOVA.

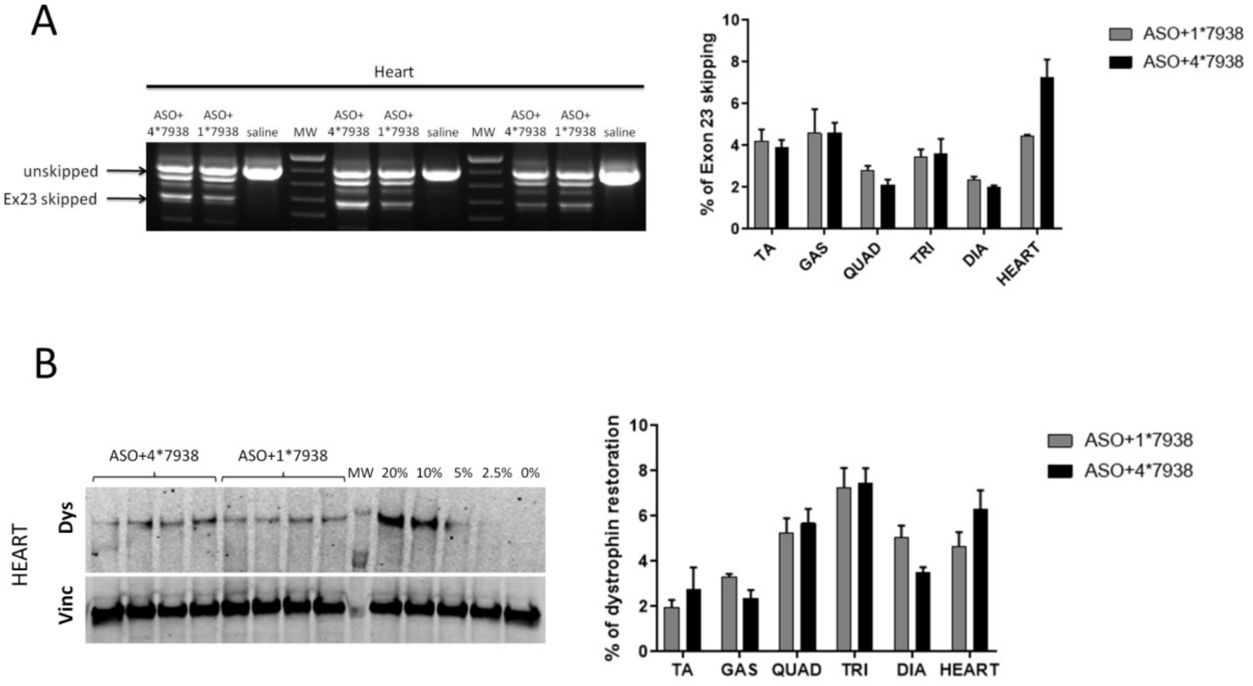

Figure S3. Comparison between 1 vs 4 injections of UNC7938 on exon skipping efficacy (A) Effect of the number of UNC7938 injections on exon skipping level. Left panel: example of the visualization of exon 23 skipping on gel in the heart of *mdx* mice treated with saline, ASO+1*7938 or ASO+4*7938. PCR amplifications between exons 20 to 26 are loaded on a 1.5% agarose gel. The top band corresponds to the unskipped transcript and the lower band to the exon 23 skipped one. Right panel: qPCR quantification of exon 23 skipping using taqman qPCR in the different muscle tissues. Tibialis anterior (TA), gastrocnemius (GAS), quadriceps (QUAD), triceps (TRI), diaphragm (DIA) and heart. Results are expressed as mean ± SEM; N=4 mice per group. P = 0.4669 between ASO+1*7938 or ASO+4*7938 analyzed by RM two-way ANOVA. (B) Quantification of dystrophin restoration level in treated *mdx* mice. A typical dystrophin western blot obtained for the heart is showed in the left panel, with vinculin used for normalization. A standard curve made from pooled lysates from C57BL10 (WT) and *mdx* control for each tissue is loaded for quantification (0%, 2.5%, 5%, 10% and 20% of WT). Right panel: quantification of dystrophin restoration using the Empiria Studio software. Results are expressed as mean ± SEM; N=3 mice per group. P = 0.7915 between ASO+1*7938 or ASO+4*7938 analyzed by RM two-way ANOVA.

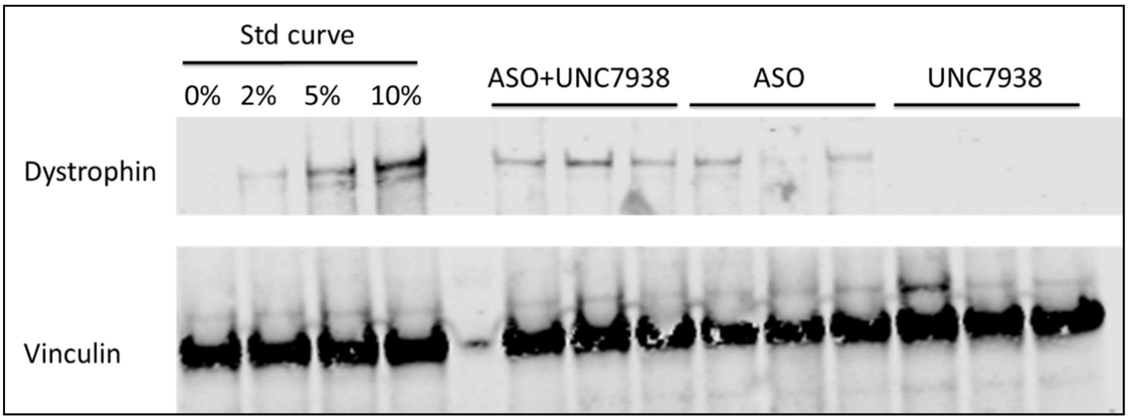

Figure S4. Treatment with UNC7938 alone has no effect on dystrophin restoration. A typical dystrophin western blot obtained for the diaphragm is showed, with vinculin used for normalization. A standard curve made from pooled lysates from C57BL10 (WT) and *mdx* control for each tissue is loaded for quantification (0%, 2%, 5% and 10% of WT).
